# 

**Published:** 1876-10

**Authors:** 


### CONTENTS.

PAGE.

**CONTRIBUTIONS.**

Use of Hypochondriacs. BEN. H. PRATT....169
What do You Charge. THOS. K. BEECHER....171
Nerve-Storms. P. H. HAYES, M. D....172
The Metric System. M. S. BIDWELL....174

**MEDICAL ITEMS AND NEWS.**

Poisoning by Belladonna—Antidote to Strychnia—Toothache—Rheumatic Mixture—Solution of Salicylic Acid—Convenient Local Anodyne—Sycosis—Pulsatilla in Amenorrhœa—Hydrocele of Infants—Santonine as a Ferbrifuge—Lime Water in Infantile Eczema—Epilepsy—Reagent for Uric Acid—Action of Strychnia on the Eye—Anæsthesia of Drunkenness—Eucalyptus Globulus—Variolus Pustules—Pityriasis—Snake Bites—Cold in the Head—Treatment of Tape Worm—Rules in Administering Arsenic—Corpulence—Puritis Vulvae—Test for Blood—Yellow Fever Poison—Acute Rheumatism—Angina Pectoris—Administering Chloroform—Aspiration in Intestinal Obstruction—Diphtheria—Bonwill's Method of Inducing Anæsthesia—Freckles—Red Faces—Nitrate of Zinc in Venereal Lesions—Salicylic Acid Peparations....176 to 184

PAGE.

**OUR CORRESPONDENCE.**

Six Letters with Replies....185

**HOUSEHOLD HINTS AND RECIPES.**

Extract of New Mown Hay—Waterproofing Goods—Vegetable Leather—To Fix Pencil Drawings—A Good Kalsomine—Cleaning Engravings—How to Build a Cistern—Cheap Paint—Preserves—Stains, to Remove....185 to 189

**EDITORIAL.**

Ethics....188
Dangerous Interference with the Ears....189
Trichina Spiralis....190

**EDITORIAL CHIT-CHAT.**

Theodore Tilton—Prince Milan—Lost Votes—Foundered—Prof. Huxley—Maj. Towner's Poem—Queer Tree—Camping Out—"Schooner"—Model City—California Jack—Campaign Character—Horrible—Spiritual Manifestations—Spectacle Venders—Centennial Exposition....192 to 197

